# Overexpression of the heavy metal-associated isoprenylated plant protein gene *IbHIPP7* reduces cadmium accumulation and alleviates cadmium toxicity in sweetpotato

**DOI:** 10.1093/hr/uhaf323

**Published:** 2025-12-08

**Authors:** Pengcheng Dong, Yumeng Yin, Shiyuan Zhang, Yujun Fan, Xinzhe Zhang, Meng Zhang, Yan Xia, Chen Chen, Liang Shi, Yahua Chen

**Affiliations:** Sanya Institute of Nanjing Agricultural University, Sanya 572024, China; College of Life Sciences, Nanjing Agricultural University, Nanjing 210095, China; Sanya Institute of Nanjing Agricultural University, Sanya 572024, China; College of Life Sciences, Nanjing Agricultural University, Nanjing 210095, China; Sanya Institute of Nanjing Agricultural University, Sanya 572024, China; College of Life Sciences, Nanjing Agricultural University, Nanjing 210095, China; Sanya Institute of Nanjing Agricultural University, Sanya 572024, China; College of Life Sciences, Nanjing Agricultural University, Nanjing 210095, China; College of Life Sciences, Nanjing Agricultural University, Nanjing 210095, China; College of Life Sciences, Nanjing Agricultural University, Nanjing 210095, China; College of Life Sciences, Nanjing Agricultural University, Nanjing 210095, China; Asian Natural Environmental Science Center, The University of Tokyo, 1-1-8 Midoricho, Nishitokyo, Tokyo 188-0002, Japan; College of Life Sciences, Nanjing Agricultural University, Nanjing 210095, China; Asian Natural Environmental Science Center, The University of Tokyo, 1-1-8 Midoricho, Nishitokyo, Tokyo 188-0002, Japan; Sanya Institute of Nanjing Agricultural University, Sanya 572024, China; College of Life Sciences, Nanjing Agricultural University, Nanjing 210095, China; National Joint Local Engineering Research Center for Rural Land Resources Use and Consolidation, Nanjing Agricultural University, Nanjing 210095, China; Sanya Institute of Nanjing Agricultural University, Sanya 572024, China; College of Life Sciences, Nanjing Agricultural University, Nanjing 210095, China; Asian Natural Environmental Science Center, The University of Tokyo, 1-1-8 Midoricho, Nishitokyo, Tokyo 188-0002, Japan; Jiangsu Collaborative Innovation Center for Solid Organic Waste Resource, Nanjing Agricultural University, Nanjing 210095, China

## Abstract

Cadmium (Cd) contamination in farmland soils poses a potential threat to crop safety and human health. Heavy metal-associated isoprenylated plant proteins (HIPPs), a unique group of proteins in vascular plants, play a crucial role in abiotic and biotic stress responses. However, their functional characterization remains limited. In this study, we identified a novel sweetpotato HIPP gene, *IbHIPP7*, and investigated its role in Cd transport and tolerance. Subcellular localization revealed that *IbHIPP7* is localized to the plasma membrane. Functional domain analysis indicated that two conserved heavy metal-associated (HMA) domains, but not the C-terminal isoprenylation motif, are essential for Cd tolerance. Transgenic sweetpotato (cultivar Sushu33) overexpressing *IbHIPP7* exhibited significantly enhanced Cd tolerance and reduced Cd accumulation in roots and shoots compared to wild-type (WT) plants. These results indicate that IbHIPP7 reduces Cd toxicity by decreasing Cd absorption and thereby enhancing Cd tolerance, providing a molecular basis for developing low-Cd-accumulating sweetpotato varieties to enhance agricultural safety.

## Introduction

Cadmium (Cd) is a widely present non-essential element but highly toxic heavy metal to both plants and animals [1]. Cd can interfere with the absorption and transport of other ions in plants, disrupt the functions of nucleic acids and proteins, increase the accumulation of reactive oxygen species (ROS), and finally lead to a series of visible plant symptoms including reduced plant height, root browning, leaf rolling, chlorosis, and necrosis [2]. Moreover, Cd can accumulate in the human body through the food chain, potentially causing health damage [3]. Thus, crop safety faces challenges.

Soil Cd concentrations vary substantially across different land use types and geographical regions. However, well-recognized benchmarks distinguishing ‘normal’ (unpolluted) and ‘Cd-polluted’ soils have been established, drawing on data from global soil surveys and national environmental quality standards [4, 5]. For typical unpolluted soils, the global background Cd concentration generally ranges from 0.01 to 0.3 mg/kg. Specifically, in agricultural soils with no anthropogenic Cd input, the average Cd level typically falls between 0.05 and 0.15 mg/kg [6]. In contrast, Cd-polluted soils are commonly defined by exceeding region-specific or international soil quality criteria. Taking China as an example, the *Environmental Quality Standard for Soils* (GB 15618-2018) classifies agricultural soils as polluted if their Cd concentration exceeds 0.3 mg/kg (for soils with pH ≤ 5.5) or 1.0 mg/kg (for soils with pH > 7.5) [7]. Globally, soils impacted by anthropogenic activities-such as mining, smelting, or long-term application of Cd-rich phosphate fertilizers-often exhibit Cd levels ranging from 1 to 100 mg/kg. In severely polluted areas, Cd concentrations can even surpass 100 mg/kg [4].

Sweetpotato (*Ipomoea batatas*), ranked the seventh most important food crop globally, also serves as a critical resource for industry and bioenergy production [8]. Among its diverse types, vegetable sweetpotatoes have been specifically bred for their nutrient-dense and palatable shoot tips, earning them esteemed titles such as the ‘Queen of Vegetables’, ‘Anticancer Vegetables’, and ‘Longevity Vegetables’ [9]. Notably, Sun *et al*. demonstrated that the leaves and shoot tips of vegetable sweetpotatoes contain higher concentrations of protein, dietary fiber, calcium, and iron compared to their storage roots [10]. However, the escalating issue of Cd pollution poses a growing threat to the safety of consuming vegetable sweetpotatoes. For Fucaishu18-a widely cultivated variety of vegetable sweetpotato-research has confirmed a positive correlation between Cd exposure levels and the accumulation of Cd in its shoots [11]. To address this risk, it is essential to screen and select sweetpotato varieties with low Cd accumulation, while also implementing strict controls to ensure the safe production of crops in Cd-contaminated areas. Prior to achieving these goals, however, a comprehensive understanding of the molecular mechanisms governing Cd absorption and transport in vegetable sweetpotatoes is prerequisite.

In recent years, the molecular mechanisms of Cd absorption and transport in higher plants, from the organ level to the cellular level, have been widely reported [12]. Genes related to Cd absorption and transport have also been identified. Among them, members of multiple gene families are involved in the absorption, transport, and accumulation of Cd in plants, including Zinc-Regulated Transporters and Iron-Regulated Transporter-like Protein (ZIP), Ca^2+^/H^+^ exchanger Antiporter (CAX), Natural Resistance-Associated Macrophage Protein (NRAMP), Multidrug and Toxic Compound Extrusion (MATE), P-type ATPase, ATP-Binding Cassette (ABC) Transporter, Low-affinity Cation Transporter (LCT), and Oligopeptide Transporter (OPT) [12]. These transporters are mostly localized on the cell membrane or vacuole membrane, regulating intercellular ion transport and intracellular ion homeostasis, respectively [13].

Heavy metal-associated isoprenylated plant proteins (HIPPs) are a group of metal chaperones that are only found in vascular plants [14–16]. HIPPs are characterized by an isoprene motif and one or two heavy metal-associated (HMA) domains, which participate in various stress responses and play an important role in detoxification of heavy metals, especially Cd [14], plant signal transduction [15], and interaction with pathogens [16]. However, few of them have been functionally reported. *Arabidopsis thaliana* triple mutants of *hipp20/21/22* are less tolerant to Cd than wild type (WT) [17]. The transcription factor MYB49 can interact with *AtHIPP44*, upregulating its transcription and subsequently reducing Cd accumulation [18]. The expression of *OsHIPP16* increased resistance to Cd in a yeast mutant [19]. High concentrations of Cd and zinc (Zn) treatment upregulated the expression of *OsHIPP29* in rice shoots and roots, while the RNAi lines showed decreased biomass compared with the WT under Cd exposure [20]. Overexpression of *HIPPI-V* exhibited enhanced Cd tolerance in wheat [21].

To date, the function of *HIPP* genes in sweetpotato remains unclarified, and there are few reports on the mechanisms underlying Cd transport in vegetable sweetpotatoes specifically. Given this research gap, exploring the molecular function of *IbHIPP* genes in sweetpotato’s response to Cd stress holds great significance for filling this knowledge void.

In our previous study, RNA sequencing (RNA-seq) was performed to identify potential genes involved in Cd response in the Fucaishu18. Among the candidate genes, the expression level of *IbHIPP7* was significantly induced by Cd treatment [22]. Building on this finding, the present study focuses on investigating the biological function of *IbHIPP7*. This work is crucial for advancing our understanding of the molecular mechanisms governing Cd response and detoxification in vegetable sweetpotatoes.

## Results

### Isolation, characterization, and phylogenetic analysis of *IbHIPP7*

In our previous study, several *HIPP* genes were identified from the Fucaishu18 cDNA library via RNA sequencing. These genes were significantly induced by Cd treatment and annotated to the metal ion transport pathway (Gene Ontology: 0030001). Among them, a unigene encoding a novel HIPP protein with two HMA domains and a C-terminal isoprene motif drew special attention, named *IbHIPP7* (accession number: SRR35395795).

The *IbHIPP7* gene consists of six introns and seven exons, encoding a 269-amino acid protein (Fig. S1). The predicted molecular weight and isoelectric point (pI) of this protein are 30.5 kDa and 5.97, respectively. Using SWISS-MODEL (with V7C775_PHAVU from *Phaseolus vulgaris* as the template, 71.04% sequence identity), its 3D structure was predicted to include α-helices (46.1%, 124 amino acids), extended strands (15.6%, 42 amino acids), β-turns (8.2%, 22 amino acids), and random coils (30.1%, 81 amino acids), distributed across the amino acid chain. Notably, its conserved HMA domains (residues 35-96 and 134-194) form a compact globular structure, with active sites centered on cysteine pairs: Cys42/Cys45 (first HMA) and Cys141/Cys144 (second HMA) (Fig. S1).

The coding sequence (CDS) of *IbHIPP7* was amplified via PCR and Sanger-sequenced, showing high consistency with NCBI entries. To study the homology and evolution, MegaBLAST alignment and phylogenetic tree construction (MEGA X, with Arabidopsis and *Oryza sativa* HIPP7 as outgroups) were performed. IbHIPP7 shared high similarity with HIPP7 from closely related species: *Ipomoea triloba* (closest), then *Ipomoea nil* (all *Convolvulaceae*). Other homologous species were mainly *Solanaceae* (Fig. S2A and B).

### Expression profiles of *IbHIPP7*

To predict the function of the *IbHIPP7* gene, we first analyzed its expression profile. The relative expression level of *IbHIPP7* in different organs was detected by Quantitative real-time PCR (qPCR). The results showed that leaves had the highest expression level among all organs (Fig. S3A), followed by petioles, stems, and roots, suggesting that it is mainly expressed in leaves.

After 48 h of gradient CdCl₂ treatment, *IbHIPP7* expression in petioles first increased then decreased with rising Cd concentration, peaking at 25 μM. No significant changes were found in leaves or adjacent stems, suggesting *IbHIPP7* may participate in Cd stress response specifically in petioles (Fig. S3B). A CdSO₄ treatment group used to exclude Cl^−^ interference showed consistent expression patterns (Fig. S3C).

### Subcellular localization of IbHIPP7

The tobacco transient expression system provides convenience for the study of subcellular localization of plant proteins [23]. To determine the localization of IbHIPP7, we instantaneously expressed green fluorescent protein (GFP)-IbHIPP7 fusion protein in tobacco leaves and cotransformed with Agrobacterium GV3101-containing marker plasmid with membrane localization signals (mCherry-PM). In addition, the epidermal cells of tobacco leaves separately transformed with the 1305: GFP empty vector and the GFP: IbHIPP7 fusion protein were subjected to plasma membrane localization using the membrane fluorescent dye FM4-64.

**Figure 1:**
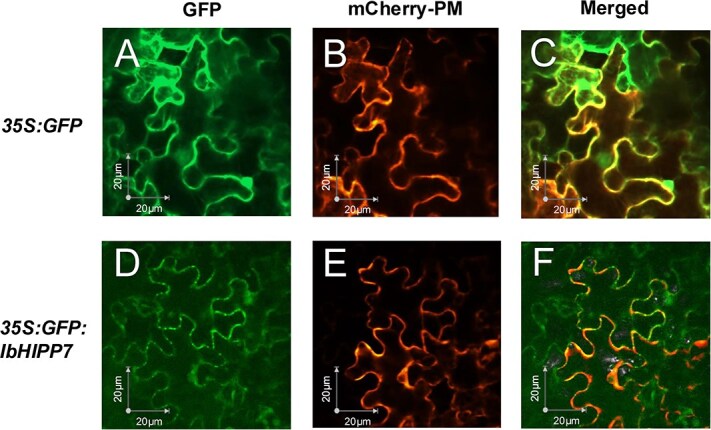
Subcellular localization of IbHIPP7 in tobacco leaves. (A) Green fluorescence of pCambia1305-35S: GFP empty vector. (B) Red fluorescence of membrane marker plasmid. (C) Merged fluorescence of A and B. (D) Green fluorescence of pCambia1305-35S: GFP: IbHIPP7 fusion. (E) Red fluorescence of membrane marker plasmid. (F) Merged fluorescence of D and E. Bars = 20 μM.

As is shown in Fig. 1 and Fig. S4, the empty vector 35S: GFP showed a diffuse distribution throughout the cytoplasm and nucleoplasm, while the GFP fluorescence of the 35S: GFP: IbHIPP7 was overlapped with the signal from the plasma membrane fluorescent marker. To further confirm the subcellular localization of IbHIPP7, green fluorescent signals were consistently detected on the cell membrane of two experimental systems: transgenic sweetpotato protoplasts and leaf epidermal cells, both of which stably expressed the GFP: IbHIPP7 fusion protein (Fig. S5). These results collectively verify that IbHIPP7 is localized to the cell membrane.

### Heterologous expression of *IbHIPP7* confers Cd tolerance in yeasts

**Figure 2:**
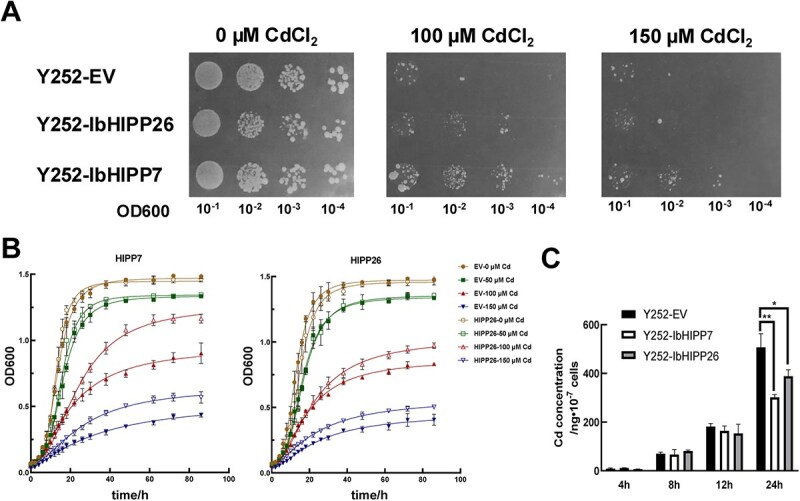
Assays of Cd detoxification activity of IbHIPPs in yeasts. (A) Yeast strain Y252 transformed with EV (pYES2 empty vector), pYES2-*IbHIPP7* and pYES2-*IbHIPP26* were grown on SD-Ura plates with indicated concentrations of CdCl_2_ for 7 days. (B) Growth curves of yeast cells expressing *IbHIPP7* and *IbHIPP26* under 0, 50, 100, and 150 μM CdCl_2_ conditions, respectively. (C) Cd accumulation in Y252-EV, Y252-*IbHIPP7*, and Y252-*IbHIPP26* yeasts. Yeast cells (1 × 10^7^) were exposed to 100-μM Cd treatment for 4, 8, 12, or 24 h.

*IbHIPP7* was heterologously expressed in yeast *Saccharomyces cerevisiae* strain Y252, with western blot confirming target protein production (Fig. S6). On SD-Ura plates with 100 μM CdCl₂, yeasts expressing *IbHIPP7* showed significantly higher Cd tolerance than empty vector (EV) controls. Arabidopsis HIPP26 (a validated Cd-related gene [24]) had a weaker tolerance-promoting effect in yeasts (Fig. 2A).

Growth curves in liquid SD-Ura medium varying CdCl₂ concentrations matched the plate results. Fifty micromolars of Cd inhibited growth but showed no difference between *IbHIPP7*-expressing yeasts and EV. The most prominent difference appeared at 100 μM Cd while higher Cd concentrations strengthened growth inhibition, reducing the difference (Fig. 2B). Collectively, these results indicate that heterologous expression of *IbHIPP7* enhances Cd tolerance in yeast cells.

Intracellular Cd concentration measurements showed *IbHIPP7*- or *IbHIPP26*-expressing yeasts accumulated significantly less Cd than EV controls, with *IbHIPP7* reducing Cd by 40%. This indicates IbHIPP7 acts as a metal chaperone to enhance Cd tolerance by lowering intracellular Cd (Fig. 2C).

### Key functional domains of IbHIPP7

**Figure 3:**
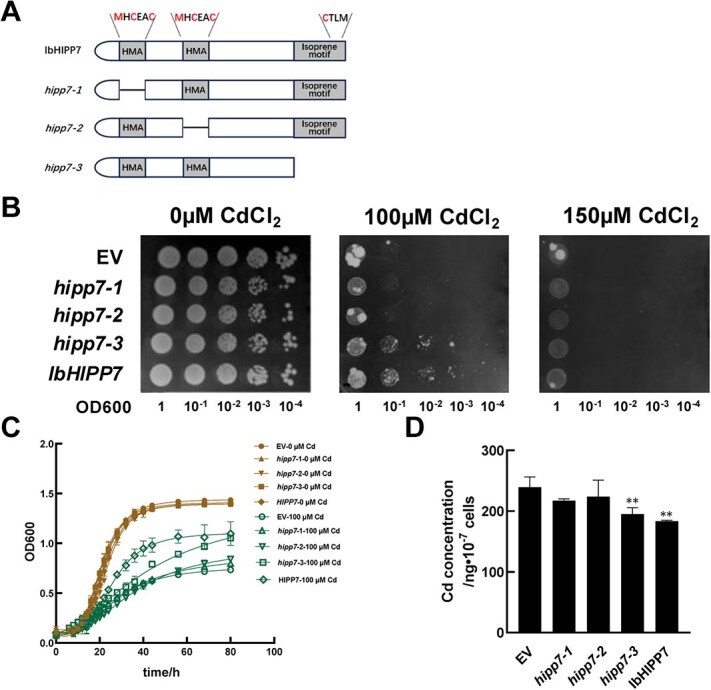
Effects of IbHIPP7 functional domain deletion on Cd tolerance in yeasts. (A) Schematic diagram of full-length CDS of IbHIPP7 and the targeted deletion sites of its functional domains. (B) Phenotypic analysis of yeast strains harboring different constructs (intact IbHIPP7, IbHIPP7 with domain deletions, or EV) grown on SD-Ura medium supplemented with the indicated concentrations of CdCl_2_ for 7 days.(C) Growth curves of yeast cells expressing different constructs under Cd-free (0 μM CdCl_2_) or Cd-stressed (100 μM CdCl_2_) conditions. (D) Cd accumulation in yeast cells treated with 100 μM CdCl_2_) for 24 h, as determined in strains harboring different constructs.

IbHIPP7 contains two HMA domains and one C-terminal isoprene motif (Fig. S1). To clarify the key functional domains underlying IbHIPP7 activity, we modified the original yeast expression vector construct YES2-IbHIPP7. Specifically, we individually knocked out the metal ion-binding sites of the two HMA domains (MHCEAC) and the C-terminal isoprene motif (CTLM) (Fig. 3A). These modified constructs, along with the original vector, were then transformed into yeast strain Y252. Plate resistance assays demonstrated that deletion of the isoprene motif had no significant impact on the Cd-tolerant function of IbHIPP7. In contrast, yeast cells lacking either of the two HMA domains completely lost Cd resistance (Fig. 3B). Yeast growth curve experiments further validated these findings. Under 100 μM Cd stress, the optical density at 600 nm (OD600) of yeasts expressing IbHIPP7 with the isoprene motif deleted was comparable to that of yeasts expressing the intact IbHIPP7 protein at the growth plateau. Conversely, yeasts transformed with the EV and those expressing IbHIPP7 lacking one HMA domain exhibited similar levels of Cd sensitivity (Fig. 3C). To assess how domain deletion affects Cd accumulation in yeasts, we quantified intracellular Cd content. The results were consistent with the plate resistance assay. Intact IbHIPP7 and isoprene-deletion yeasts accumulated less Cd than EV. In contrast, yeasts lacking either HMA domain showed no significant difference in Cd accumulation from EV (Fig. 3D). Collectively, these results confirmed that the two HMA domains are essential for the Cd-tolerant function of IbHIPP7.

### Specificity of IbHIPP7 in metal chelation and detoxification

Given that most metal transporters exhibit low substrate specificity, we further investigated the chelating specificity and detoxification capacity of IbHIPP7 toward different metal ions. Cd^2+^ frequently enters cells via transporters or ion channels for copper (Cu^2+^), manganese (Mn^2+^), Zn^2+^, and iron (Fe^2+^)-a phenomenon attributed to the similar chemical properties of these divalent cations, including comparable ionic radii and shared transition metal electron configurations.

Our results showed that yeast cells inherently displayed significantly higher tolerance to Cu^2+^, Mn^2+^, Zn^2+^, and Fe^2+^ than to Cd^2+^ (Fig. S7). Notably, however, expression of *IbHIPP7* failed to mitigate the toxicity of excess Cu^2+^, Mn^2+^, Zn^2+^, or Fe^2+^ to yeast cells. This lack of cross-detoxification was further confirmed by phenotypic analysis of IbHIPP7 functional domain deletion mutants, which also showed no rescue of yeasts growth under excess concentrations of these four metals.

Collectively, these findings demonstrate that IbHIPP7 exhibits distinct substrate specificity, functioning specifically in the chelation and detoxification of Cd^2+^ rather than other structurally similar divalent transition metals.

### *IbHIPP7* overexpression enhances Cd tolerance in Arabidopsis

Previous studies have established the cytological role of *IbHIPP7* in Cd detoxification. To further explore its biological function in plants, we generated three T3 transgenic Arabidopsis lines overexpressing *IbHIPP7* (OE lines) using agrobacterium-mediated floral dip transformation. The positive transgenic plants were confirmed by qPCR (Fig. S8A) and western blot analysis (Fig. S8B).

**Figure 4:**
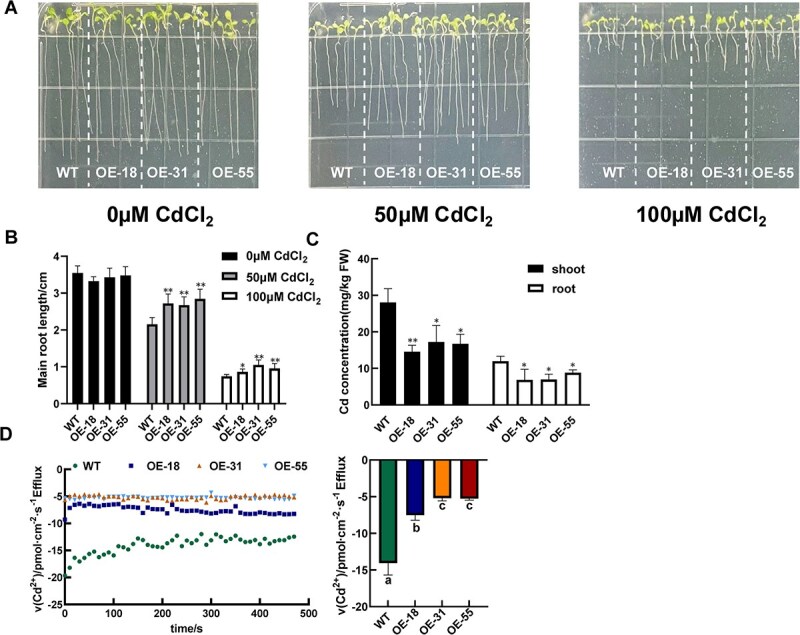
Phenotypic and physiological responses of *IbHIPP7*-expressing Arabidopsis to Cd stress. (A) and (B) Primary root length analysis of WT (Col-0) and *35S: GFP: IbHIPP7* transgenic lines. Plants were grown on one-half MS agar plates supplemented with 0, 50, or 100 μM CdCl_2_ for 7 days, with root lengths measured to assess Cd-induced growth inhibition. (C) Cd concentration in shoots and roots of Arabidopsis. Two-week-old seedlings were first acclimated in one-fourth-strength Hoagland solution for 1 week, then treated with 5 μM CdCl_2_ for another 7 days. Shoots and roots were harvested separately, and Cd content was determined by ICP-MS. Each biological replicate consisted of three seedlings. (D) Net Cd^2+^ flux in root segments of WT (green dots) and *IbHIPP7*-OE lines (OE-18, dark blue dots; OE-31, orange dots; OE-55, light blue dots). Fluxes were measured in 5-mm-long root segments at a position 300 μm from the root tip. All plants were incubated in a measuring solution containing 0.1 mM KCl, 0.01 mM CdCl_2_, and 0.3 mM 2-(N-morpholino)ethanesulfonic acid (MES) (pH 5.6) during the assay.

When grown on Cd-free agar plates, no significant differences in primary root growth were observed between WT plants and *IbHIPP7*-OE lines. As Cd concentrations increased, root growth was progressively inhibited, and phenotypic differences became evident: OE lines exhibited significantly greater Cd tolerance compared to WT plants (Fig. 4A and B). To investigate the basis of this enhanced tolerance, we measured Cd accumulation in WT and transgenic plants. Both shoots and roots of *IbHIPP7*-OE lines contained significantly lower Cd concentrations than those of WT plants (Fig. 4C). To determine whether this reduced accumulation stemmed from differences in root Cd uptake rates, we measured Cd^2+^ flux at the root surface of OE lines and WT plants exposed to 5 μM CdCl₂. Cd^2+^ uptake was most rapid at ~300 μm from the root cap, with WT plants exhibiting a significantly higher absorption rate than OE lines (Fig. 4D). These findings collectively indicate that overexpression of *IbHIPP7* enhances plant tolerance to Cd by reducing Cd accumulation, likely through decreased root uptake of Cd^2+^.

### *IbHIPP7* overexpression inhibited the uptake and accumulation of Cd by the roots of sweetpotatoes

To investigate the function of *IbHIPP7* in Cd uptake by sweetpotato roots, we first generated transgenic hairy roots via a novel *Agrobacterium rhizogenes*-mediated rapid genetic transformation method [25]. We then directly assessed *IbHIPP7*’s role in Cd handling through hydroponic experiments. Following a 1-week treatment with 5 μM CdCl₂, the Cd concentration in hairy roots overexpressing *IbHIPP7* was significantly lower than that in hairy roots of the WT sweetpotato cultivar Fucaishu18 (Fig. S9C). To further confirm whether *IbHIPP7* overexpression directly inhibits root Cd uptake, we employed Noninvasive Microtest Technology (NMT) to measure Cd^2+^ flux at the sweetpotato root surface. The results showed that the Cd^2+^ influx rate at the root surface of *IbHIPP7*-OE lines was significantly reduced compared to that of the WT (Fig. S9A and B).

### Functional validation of *IbHIPP7* in transgenic sweetpotato (Sushu33) at the whole-plant level

**Figure 5:**
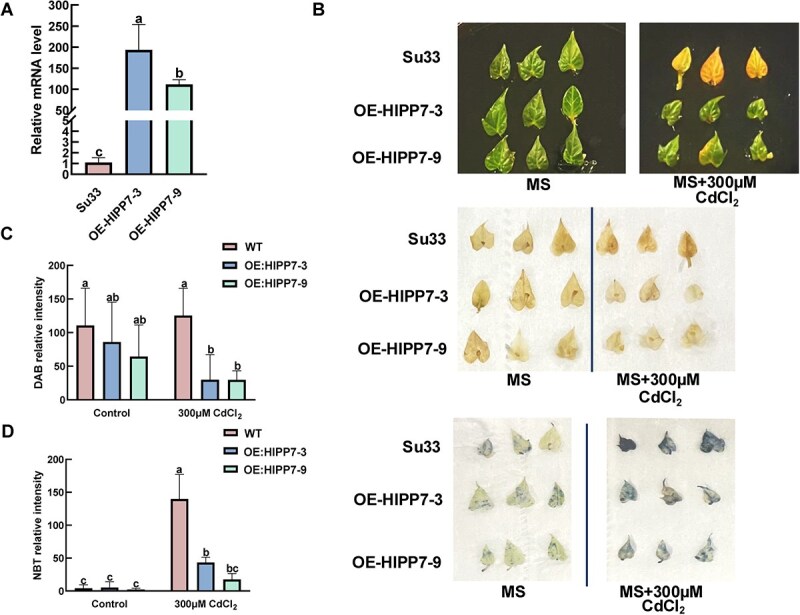
Phenotype of transgenic leaves of Sushu33 after 0 and 300 μM CdCl_2_ treatment. (A) Relative mRNA expression levels of *IbHIPP7* in WT Sushu33 and transgenic lines (OE-HIPP7-3, OE-HIPP7-9), determined by qPCR using the 2^-ΔΔCt^ method. (B) *In vitro* leaf phenotype of WT Sushu33 and two *IbHIPP7*-overexpression lines. Leaves were cultured on MS medium with 0 μM (Cd-free control) or 300 μM CdCl₂ for 1 week. (C) DAB staining of detached leaves from WT and *IbHIPP7*-overexpression lines, used to detect hydrogen peroxide (H₂O₂, a type of ROS) accumulation under Cd stress. (D) NBT staining of detached leaves from WT and *IbHIPP7*-overexpression lines, used to detect superoxide anion (O₂^−^, a type of ROS) accumulation under Cd stress.

Given the potential for *IbHIPP7* to be predominantly expressed in leaves, we further confirmed its biological function in sweetpotato at the whole-plant level by generating *IbHIPP7*-OE lines of the cultivar Sushu33 via traditional *Agrobacterium tumefaciens*-mediated genetic transformation. PCR and western blot analysis confirmed the successful expression of the GFP-IbHIPP7 fusion protein (~57 kDa) in the leaves of transgenic sweetpotato lines (Fig. S10A and B). qPCR experiment further showed that the *IbHIPP7* expression levels in the OE lines (OE:HIPP7-3 and OE:HIPP7-9) were 100-200 times higher than those in WT Sushu33 (Fig. 5A); these two OE lines were selected for subsequent phenotypic validation.

*In vitro* leaf experiments revealed no significant phenotypic differences between WT Sushu33 and transgenic sweetpotato leaves when grown on Murashige and Skoog (MS) medium without Cd for 1 week. However, under treatment with 300 μM CdCl₂, WT leaves almost completely yellowed, whereas *IbHIPP7*-OE lines retained green pigmentation. This indicated that *IbHIPP7* overexpression enhanced the Cd tolerance of sweetpotato leaves (Fig. 5B). Since Cd stress induces the accumulation of ROS [2], we performed 3,3′-diaminobenzidine (DAB) and nitroblue tetrazolium (NBT) staining on detached leaves to verify whether *IbHIPP7* overexpression alleviates Cd-induced ROS damage (Fig. 5C and D). The staining results showed that leaves of *IbHIPP7-*OE sweetpotato accumulated significantly less ROS than those of WT Sushu33, confirming the protective effect of *IbHIPP7* against Cd toxicity.

**Figure 6:**
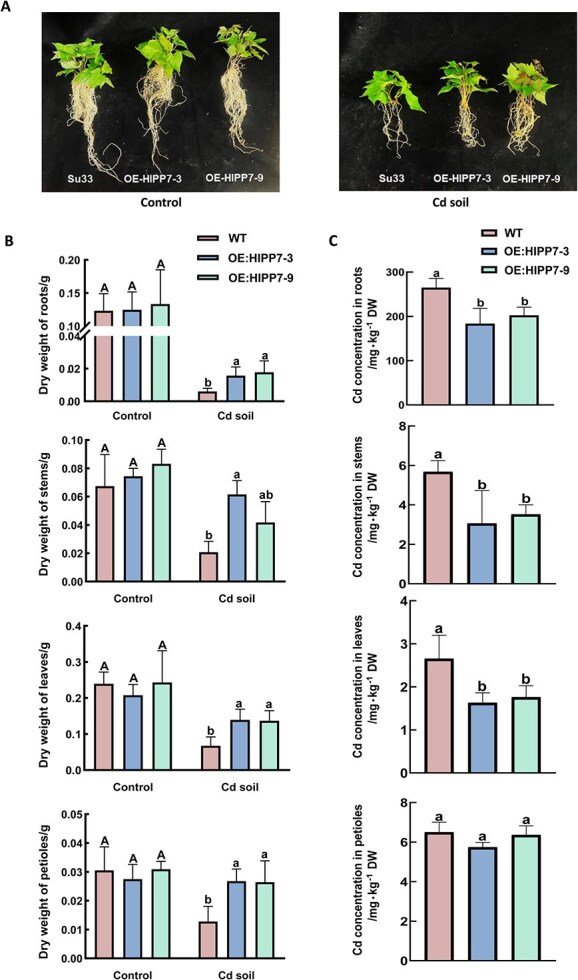
Pot experiment of sweetpotatoes in Cd-contaminated soils. (A) Sweetpotato cultivar Sushu 33 (WT) and two *IbHIPP7*-OE lines were planted in Cd-contaminated soils (5.3 mg/kg Cd) and control soils (non-Cd-contaminated soils) and sampled after 45 days of growth. (B) Dry weight of roots, stems, and leaves of different sweetpotato lines (WT and transgenic lines). (C) Cd concentration in roots, stems, leaves, and petioles of different sweetpotato lines (WT and transgenic lines).

To clarify the role of *IbHIPP7* overexpression in Cd accumulation in sweetpotatoes, a pot experiment was performed using Cd-contaminated soils in this study. The results indicated no significant difference in the biomass of roots, stems, leaves, and petioles between WT and transgenic sweetpotatoes grown in control soils (non-Cd-contaminated soils). In contrast, when cultivated in Cd-contaminated soils, the biomass of roots, stems, leaves, and petioles of *IbHIPP7*-OE sweetpotatoes was significantly higher than that of the WT (Fig. 6A and B). This finding suggests that *IbHIPP7* overexpression enhances Cd tolerance in sweetpotatoes.

Measurements of Cd concentrations in different sweetpotato organs showed that, compared with the WT, the Cd concentrations in roots, stems, and leaves of transgenic sweetpotatoes were reduced by 28%, 42%, and 38%, respectively. Notably, no significant difference in petiole Cd concentration was observed between the two groups (Fig. 6C). Given that previous studies have demonstrated Cd-induced upregulation of *IbHIPP7* in petioles, this study further analyzed the proportion of Cd content in stems, leaves, and petioles relative to the total Cd content in aboveground tissues. The results revealed that the proportion of Cd content in petioles of *IbHIPP7*-OE sweetpotatoes was significantly higher than that of the WT (Fig. S11). This observation implies that *IbHIPP7* may modulate the translocation and reallocation of Cd in aboveground parts of sweetpotatoes by regulating the physiological functions of petioles.

## Discussion

Sweetpotato (*I. batatas*), a globally important root crop, is unique in that both its stem tips and storage roots are edible [9]. A comparison of Cd content across different sweetpotato tissues reveals that the absorption and accumulation of Cd in stems and leaves are significantly higher than those in storage roots [26]. Consequently, the health risk associated with consuming sweetpotato stems and leaves is greater than that of consuming storage roots. Furthermore, substantial variations in health risks from ingesting stems, leaves, and storage roots have been observed among different sweetpotato cultivars [27]. For this reason, special attention must be paid to the safe production of sweetpotato shoots-particularly for cultivars grown specifically as leafy vegetables. Cd is absorbed by plant roots and then transported to different tissues and organs. Notably, it can be efficiently transported from senescing tissues to young tissues via the phloem, facilitating Cd redistribution throughout the plant [28]. Within a specific concentration range, plants can activate intrinsic detoxification mechanisms to mitigate Cd toxicity. Current studies have identified that the primary plant response mechanisms to mitigate Cd toxicity are root retention, antioxidant system activation, chelation, osmoregulation, and compartmentalization [29]. A large number of genes are involved in these processes, including those encoding ROS scavenging enzymes, phytohormone regulators, metal transporters, and osmotic adjustment proteins. Among these, metal transporters directly mediate Cd absorption, translocation, and chelation [12].

HIPPs are a class of metallochaperones critical for the safe intracellular transport of metal ions [30]. However, many plant heavy metal transporters remain uncharacterized at the molecular level, with their transport efficiency, substrate specificity, and subcellular localization still unclear. In our previous work, *IbHIPP7* was identified as a Cd-inducible gene through transcriptome analysis of Fucaishu18. Sequence analysis and functional prediction suggested that *IbHIPP7* may play roles in Cd response and detoxification.

Yeasts are widely used to characterize the cellular functions of plant transporter genes, as it shares core eukaryotic organelles with plants such as cell walls and vacuoles. For instance, overexpression of tea plant (*Camellia sinensis*) genes *CsHIPP22*, *CsHIPP24*, and *CsHIPP36* rescued the Cd-sensitive yeast mutant *ycf1* and enhanced yeast tolerance to Cd stress [31]. Knah *et al*. found that the expression of rice *OsHIPP16* improved yeast detoxification of Cd and Zn but had no effect on tolerance to Mn or Cu stres [19]. In contrast, transgenic yeasts expressing *OsHIPP17* exhibited significantly lower Cd tolerance compared to yeasts harboring an EV [32]. These findings indicate that different members of the same gene family may exert opposing functions. In our study, yeasts expressing sweetpotato *IbHIPP7* showed significantly higher Cd tolerance than yeasts carrying an EV or expressing *IbHIPP26*-confirming our hypothesis that *IbHIPP7* acts as a Cd toxicity alleviator.

Currently, critical questions remain unresolved: the nature of the interaction between these transporters and metal ions, and the relationship between transporter structure and function. Answering these questions is essential to elucidating the molecular mechanisms of Cd uptake and translocation in plants [33]. Known metal ion transporter families typically contain conserved metal-binding domains (MBDs)-a feature supported by protein domain datasets (predicted via HMMER software) and phylogenetic analyses (using data from the Pfam database) for each family [34]. Most HMA transporters within a given family possess either one or two predicted MBDs, reflecting high sequence conservation. MBDs are enriched in amino acids such as cysteine (Cys), methionine (Met), glutamic acid (Glu), aspartic acid (Asp), and histidine (His)-residues with sulfur (S), nitrogen (N), or oxygen (O) atoms that can act as electron donors to bind metal ions. The motif M/LXCXXC (where M = Met, L = Leucine, C = Cys, and X = any amino acid) has been confirmed as the core of the HMA domain, with affinity for Cu^2+^, Zn^2+^, and nickel (Ni^2+^) [35]. Our analysis revealed that the IbHIPP7 protein contains two HMA domains, each with the identical MBD sequence ‘MHCEAC’-a sequence consistent with the conserved M/LXCXXC core motif. Notably, deletion of either HMA domain abolished IbHIPP7-mediated Cd tolerance in yeasts, indicating that the two domains are not functionally redundant. In contrast, the related functional protein IbHIPP26 contains only one HMA domain and has a molecular weight approximately half that of IbHIPP7. We further investigated IbHIPP7’s chelating and detoxifying activity toward multiple metal cations (Cu^2+^, Zn^2+^, and Fe^2+^) but observed no significant binding to these ions. This suggests that different HIPP proteins may exhibit substrate specificity in metal ion chelation and transport.

Protein isoprenylation is a post-translational lipid modification process, wherein a farnesyl (15-carbon) or geranylgeranyl (20-carbon) isoprene group is covalently attached to conserved Cys residues at or near the protein’s C-terminus. Research has shown that the biological function of many proteins depends heavily on their membrane localization-and post-translational isoprenylation is often a prerequisite for this localization [36]. For example, newly synthesized, unmodified Rab proteins are dispersed in the cytoplasm and functionally inactive; however, following isoprenylation, Rab proteins localize to specific organelle membranes (with the assistance of other proteins) and perform vesicle transport functions [37]. Our data demonstrated that IbHIPP7 alleviates Cd toxicity in yeast cells, but the role of isoprenylation in IbHIPP7 localization and function remained unknown. To address this, we investigated the impact of the isoprenylation motif on IbHIPP7 function. Surprisingly, deletion of this conserved motif had almost no effect on IbHIPP7-mediated Cd tolerance in yeasts. This suggests that while the isoprenylation motif is conserved in IbHIPP7, it is not required for its Cd tolerance function.

Existing studies have proposed a working model for HIPP function: HIPPs bind toxic heavy metal ions or excess essential metal ions via their HMA domains, transfer these ions to metal transporters, and maintain intracellular ion homeostasis through either cell membrane efflux (transporting ions out of the cell) or vacuolar sequestration (trapping ions in vacuoles) [38]. For example, the rice metallochaperone OsATX1 binds Cu^2+^ and transfers it to metal transporters such as HMA4-6 and HMA9 [39]. Unlike metal transporters, metallochaperones like HIPPs exhibit diverse subcellular localizations, enabling them to perform distinct roles in regulating plant heavy metal tolerance [40]. Our results showed that IbHIPP7 localizes to the cell membrane and enhances Cd tolerance in both yeasts and Arabidopsis while reducing Cd accumulation. In sweetpotato, overexpression of *IbHIPP7* significantly decreased Cd concentrations in roots and shoots. Based on these findings, we hypothesize that *IbHIPP7* directly or indirectly inhibited Cd absorption thus contributing to Cd detoxification.

Notably, the overexpression of the *IbHIPP7* gene did not significantly reduce Cd concentration in the petioles of sweetpotatoes. The vascular system of the petiole serves as a critical passageway for Cd, where the xylem is responsible for upward transport and the phloem enables bidirectional transport [41]. Specifically, this system mediates the movement of Cd from the roots to the leaves on one hand, and participates in the redistribution of Cd from the leaves to other organs on the other hand [28]. By regulating the transport flux and transport direction of Cd within the vascular tissues, the petiole helps balance the overall Cd accumulation level of the plant [42]. Through the analysis of the *IbHIPP7* expression profile, we found that the expression level of *IbHIPP7* in sweetpotato petioles was significantly increased under Cd induction, suggesting that *IbHIPP7* is likely to regulate the redistribution of Cd in plants through the petiole. A key piece of evidence is that although overexpression of *IbHIPP7* did not change the Cd concentration in the petioles, the proportion of Cd content in the petioles relative to the total Cd content in the entire aboveground parts was significantly increased.

In conclusion, this study uncovers a novel function of the *IbHIPP7* gene in mediating Cd response and detoxification in sweetpotato. Overexpression of *IbHIPP7* reduced Cd concentrations in sweetpotato roots, stems and leaves by 28%, 42%, and 38%, respectively. These findings provide valuable insights into the molecular mechanisms of Cd transport in sweetpotato and identify a key gene for improving sweetpotato quality and ensuring its safe production-particularly in Cd-contaminated agricultural soils.

## Materials and methods

### Plant materials and treatment

Virus-free seedlings of the vegetable-type sweetpotato cultivar Fucaishu18 were used for gene expression analysis, detoxified via sweetpotato meristem-tip culture [43]. Fifteen-centimeter-long stem tips from seedlings grown in a 6:3:1 (vermiculite: nutritional soil: perlite) substrate were transferred to 400 ml black plastic bottles with one-half Hoagland solution (pH 5.8) for hydroponics. The nutritional soils were purchased from Huai'an Huisheng Agriculture, Forestry and Horticulture Development Co., Ltd, China. Seedlings were maintained in a growth chamber (27°C/25°C day/night, 70% RH, 12 h light/12 h dark, 400 μmol·m^−2^·s^−1^ light intensity), with nutrient solution refreshed every 2 days [44]. After 2 weeks of cultivation, the third fully expanded leaf (petiole excluded) of each seedling was immersed in 0 (control), 10, 25, 50, or 100 μM CdCl₂ solutions for 1 min. Leaves were removed from the solution and air-dried to form a uniform water film, and blotted to remove excess solution. The control group was treated identically with deionized water. At 48 h post-treatment, treated leaves, petioles of treated leaves and stems were collected and immediately frozen in liquid nitrogen and stored for RNA extraction.

### Cloning of the coding sequence of *IbHIPP7*

All primer designs for gene amplification were based on the genomic sequence of the sweetpotato cultivar Taizhong 6 (assembly accession: GCA_002525835.2) available in the NCBI Genome database. RT-PCR was used to amplify the CDS of *IbHIPP7* (primer pair IbHIPP7-F/R, Table S1). PCR products were purified using the MolPure® Gel Extraction Kit (Yeasen, China) and then cloned into Hieff Clone® Zero TOPO-Blunt Simple Vector (Yeasen, China). Positive clones were sent to Sangon Biotech (China) for Sanger sequencing. Four independent clones were sequenced to confirm the accuracy of the IbHIPP7 CDS.

### Bioinformatics analysis of IbHIPP7 protein

Conserved domains of IbHIPP7 protein were analyzed using the online database PROSITE (https://prosite.expasy.org/). 3D structure prediction was generated using the SWISS-MODEL server (http://www.swissmodel.expasy.org). Multiple sequence alignment and phylogenetic analysis were carried out using MEGA-X software. Homologous protein sequences of IbHIPP7 were retrieved from the NCBI database (https://www.ncbi.nlm.nih.gov/) via BLAST searches. A Maximum Likelihood (ML) phylogenetic tree was constructed with 1000 bootstrap replicates to assess node reliability. The predicted chemical and physical parameters of IbHIPP7 protein such as amino acid composition, the molecular weight, theoretical pI, and atomic composition were calculated by ProtParam tool (https://web.expasy.org/protparam/).

### RNA extraction and gene expression analysis

Stems, leaves, and petioles of Fucaishu18 were used for RNA extraction. Total RNA was isolated using the SteadyPure Plant RNA Extraction Kit (Accurate Biotechnology, China) following the manufacturer’s protocol. First-strand cDNA was synthesized from 1 μg of total RNA using the Evo M-MLV RT Mix Kit (Accurate Biotechnology, China); this cDNA served as the template for both CDS amplification and gene expression analysis. For qPCR analysis, reactions were performed using a QuantStudio™ 5 Real-Time PCR System (Thermo Fisher Scientific, Waltham, MA, USA). The housekeeping gene *IbActin* was used as the internal reference to normalize gene expression levels. Each 20 μl reaction mixture contained 10 μl of 2 × SYBR Green Premix (Accurate Biotechnology, China), 0.8 μl of each forward and reverse primer (10 μM), 1 μl of diluted cDNA template (50 ng/μl), and 7.4 μl of nuclease-free water. The thermal cycling conditions were: Initial denaturation at 95°C for 3 min; 40 cycles of denaturation at 95°C for 15 s, annealing and extension at 60°C for 30 s; a final melting curve analysis (65°C-95°C, increment 0.5°C every 5 s) to confirm amplicon specificity. Relative gene expression levels were calculated using the 2^-ΔΔCt^ method [45], with three biological replicates and three technical replicates per sample to ensure reproducibility.

### Subcellular localization of IbHIPP7

The coding sequence of *IbHIPP7* was fused to the C-terminus of GFP without stop codon in the expression vector pCambia1305 (primer pair GFPIbHIPP7-F/R, Table S1) using Hieff Clone® Plus One Step Cloning Kit (Yeasen, China) and the construct was introduced into *A. tumefaciens* strain GV3101. The infection method was described as Yu *et al*. [46]. After 2 days of infection, the tobacco leaves were recovered and sampled. Confocal imaging analysis was performed using Ultra View Vox system (PerkinElmer, USA). For GFP, an excitation filter with a 480- to 500-nm bandpass and an emission filter with a 510- to 550-nm bandpass were used. For Red Fluorescent Protein (mCherry), an excitation filter with a 540- to 560-nm bandpass and an emission filter with a 570- to 620-nm bandpass were used.

### Heterologous expression in yeasts

The *IbHIPP7* CDS was inserted into the yeast expression vector pYES2 (downstream of the GAL1 promoter) between the XbaI and BamHI restriction sites using primer pair YES2IbHIPP7-F/R (Table S1). To generate deletion mutants lacking conserved domains, modified vectors were constructed via homologous recombination with primer pairs Mut1 ~ 3-IbHIPP7-F/R (Table S1).

All vectors (empty pYES2 as control, pYES2-*IbHIPP7*, and mutant constructs) were transformed into *S. cerevisiae* strain Y252 using the lithium acetate method. Positive transformants were selected on uracil-deficient synthetic dropout medium (SD-Ura). For the plate growth assay, yeast cells were precultured in liquid SD medium containing 2% glucose. A one-one thousandth volume of the preculture was transferred to liquid SD-Ura medium supplemented with 2% galactose and cultured until OD600 reached 1.0. Cell suspensions were serially diluted to OD600 values of 0.1, 0.01, 0.001, and 0.0001, then spotted onto solid SD-Ura medium (with 2% galactose) containing 0, 100, or 150 μM CdCl₂. Plates were incubated at 30°C for 7 days and photographed to assess growth. To further investigate the growth rate of yeast cells expressing *IbHIPP7* gene or not (EV) exposed to Cd treatment, the growth curves of yeast cells were detected after Cd exposure. One milliliter of yeast solution with an OD600 value of ~1.0 was taken and diluted with 20 ml of SD-Ura liquid medium containing 0, 50, 100, and 150 μM CdCl_2_, respectively. At 8, 10, 12, 14, 16, 18, 20, 22, 24, 26, 28, 32, 36, 40, 44, 48, 60, 72, 84, and 96 h after Cd treatment, 2-ml aliquots of the yeast suspensions were collected to determine the OD600 values, and a yeast growth curve was plotted.

### Heterologous expression in Arabidopsis

The pCambia1305-35S: GFP: IbHIPP7 construct was transformed into *A. tumefaciens* strain GV3101. Arabidopsis plants were transformed via the floral dip method [47]. Transgenic lines were initially selected based on hygromycin resistance and further verified by qPCR. Homozygous T3 generation lines were used for subsequent experiments. Arabidopsis seeds were sterilized by soaking in 75% ethanol for 1 min, followed by immersion in 10% sodium hypochlorite for 15 min. Seeds were rinsed with sterile deionized water and sown on one-half MS solid medium (supplemented with 30 g/l sucrose) containing 0, 50, or 100 μM CdCl₂. After 2 days of cold stratification (4°C), plates were transferred to a light incubator (23°C, 16 h light/8 h dark) for 1 week. The length of the primary root was measured to evaluate Cd tolerance. For the Cd accumulation assay, 3-week-old Arabidopsis seedlings (WT and transgenic lines) were transferred to hydroponic cups containing one-fourth Hoagland nutrient solution (three seedlings per cup) and acclimated for 1 week. Seedlings were then treated with 5 μM CdCl₂ for 2 weeks. Each treatment group included three biological replicates. After treatment, seedlings were harvested for Cd concentration analysis.

### *Agrobacterium rhizogenes* infection and hydroponic experiments

The pCambia1305-35S: GFP: IbHIPP7 construct was transformed into *A. rhizogenes* strain K599 to induce Fucaishu18 transgenic hairy roots. Seedlings with hairy roots were acclimated in hydroponics for 1 week, then treated with one-fourth Hoagland +5 μM CdCl₂ for 1 week. Root samples were collected post-treatment and split into two portions: one part for NMT-based Cd^2+^ flux measurement; the other part washed with deionized water, soaked in 10 mM EDTA-2Na for 15 min (to remove apoplastic Cd), rinsed three times with deionized water, dried, and analyzed for Cd content.

### Generation of transgenic sweetpotato overexpressing *IbHIPP7*

The pCambia1305-35S: GFP: IbHIPP7 construct was introduced into *A. tumefaciens* strain EHA105, which was used to transform the sweetpotato cultivar Sushu33 for phenotypic analysis. Transgenic sweetpotato plants were selected based on hygromycin resistance and validated by PCR, qPCR, and western blot analysis.

### Cd resistance test of transgenic sweetpotato leaves *in vitro*

The second fully expanded leaves of sterile transgenic and WT seedlings were excised, and its petiole was inserted into MS medium containing 0 or 300 μM CdCl₂. Phenotypes were recorded after 1 week of incubation.

For detection of reactive ROS, leaves were stained with DAB and NBT (Shanghai Yuanye Biotechnology Co., Ltd, China). Briefly, leaf samples were immersed in DAB or NBT staining solution for 6 h, then decolorized in 95% ethanol for 24 h to visualize ROS accumulation. Staining intensity was quantified using ImageJ software.

### Pot experiment of transgenic sweetpotatoes for phenotype analysis

Cd-contaminated soils were collected from the vicinity of a mining area (28° N, 114° E) in Changsha, Hunan Province, China. Its physicochemical properties were as follows: pH 5.46, organic matter content 32.7 g/kg, organic carbon content 19.0 g/kg, total nitrogen content 1.68 g/kg, hydrolyzable nitrogen content 196 mg/kg, total phosphorus content 0.17%, available phosphorus content 5.7 mg/kg, total potassium content 18.1 g/kg, and available potassium content 157 mg/kg. The total Cd concentration in the soils was 5.3 mg/kg. Non-Cd-contaminated soils were used as control soils. Shoot tips (containing two leaves and one terminal bud) of WT Sushu33 and two transgenic lines were planted in pots filled with either Cd-contaminated soils or control soils. Plants were grown in a light incubator (27/25°C day/night, 70% relative humidity, 12 h light/12 h dark, 400 μmol·m^−2^·s^−1^ light intensity) for 1 month. Roots, stems, and leaves were harvested separately, rinsed with deionized water, and dried. Dry weights were recorded, and samples were digested with a mixture of HNO₃ and HClO₄ (4:1, v/v) for subsequent element content analysis.

### Determination of metal concentration

The plant roots were washed with 20 mmol/l EDTA-2Na solution and deionized water for three times to remove the metal ions in the intercellular space. Then, roots and shoots were weighed and collected separately and dried at 60°C thoroughly. About 0.2 g of dry samples were wet-digested with 5 ml HNO_3_. Digestion solutions were diluted with deionized water with a ratio of 1: 10. Subsequently, the Cd concentration was detected by Inductively Coupled Plasma Mass Spectrometry (ICP-MS, NexION 2000, PerkinElmer).

For the yeast cells, 100 μM CdCl_2_ was added to the medium and the cells were incubated for 4, 8, 12, and 24 h. The yeast cells were washed with deionized water for three times and then dried at 60°C overnight. The digestion and determination methods were the same as above.

### Measurement of net Cd^2+^ flux

Net Cd^2+^ flux at the root tip was measured using NMT for real-time detection of ion/molecule transport across living cell membranes-with an NMT150-series system (Younger USA LLC, USA). In brief, Cd ion exchanger (XY-SJ-Cd) was filled with 10 mM Cd(NO₃)₂ + 0.1 mM KCl (50 μM length), equilibrated in basic salt medium (0.05 mM CdCl₂, 0.1 mM KCl, 0.3 mM MES, pH 5.6) for 0.5 h, and calibrated (50/5 μM Cd^2+^; Nernstian slope: 25-32 mV, *R*^2^ > .999). Flux was measured at 300 μm from root cap in 10 μM CdCl₂.

### Statistical analysis

All data were derived from at least three replicates. Data were processed using Microsoft Excel and plotted by GraphPad Prism 8.0. Analysis of variance (ANOVA) was performed with SPSS 19.0. The LSD test was used to explain significant differences between treatments, (P < 0.05, *; P < 0.01, **). Staining intensity was analyzed by Image J software.

## Supplementary Material

Web_Material_uhaf323

## Data Availability

The data supporting the results of this work can be obtained in the paper and its supplementary materials. The genomic data used in this study, derived from the sweetpotato cultivar Taizhong6, can be accessed in the NCBI Genome database under the assembly accession number GCA_002525835.2. https://www.ncbi.nlm.nih.gov/genome/?term=GCA_002525835.2
