# Supplementary material for: Overexpression of the heavy metal-associated isoprenylated plant protein gene IbHIPP7 reduces cadmium accumulation and alleviates cadmium toxicity in sweetpotato
Source: Hortic Res. 2025 Dec 8;13(3):uhaf323. doi: 10.1093/hr/uhaf323 (PMC12981327; doi:10.1093/hr/uhaf323)
Supplement: Web_Material_uhaf323 [file web_material_uhaf323.zip › Main_Document-Article_(clean).docx]

**Supplemental materials**

Table. S1. Primers used for gene cloning.

| IbHIPP7-F | CACAGTGAACTCTTCCAT |
| --- | --- |
| IbHIPP7-R | AACTACATCTGCCAACTC |
| GFPIbHIPP7-F | TGGACGAGCTGTACAGATCTATGGGTGAGAAAGGGGAAG |
| GFPIbHIPP7-R | TGGTCACCAATTCACACGTGTTACATGAGGGTGCAAGCGT |
| YES2IbHIPP7-F | ACCGAGCTCGGATCCATGGGTGAGAAAGGGGAAG |
| YES2IbHIPP7-R | ATGCGGCCCTCTAGATTACATGAGGGTGCAAGCG |
| Mut1-IbHIPP7-F | GGTTGATGCTAGAAAAGTCACAAGATC |
| Mut1-IbHIPP7-R | TTTTCTAGCATCAACCTTGAGCACAAT |
| Mut2-IbHIPP7-F | GTTCAAGCTCAGGTCTTGCAGAAGCGA |
| Mut2-IbHIPP7-R | GACCTGAGCTTGAACTTTCAGAACGACT |
| Mut3-IbHIPP7-F | CTCACGCTTAATCTAGAGGGCCGCATCA |
| Mut3-IbHIPP7-R | CTAGATTAAGCGTGAGGGTTCTCGTCG |
| qIbHIPP7-F | AGGGAAGGAGGGTGAGAAGA |
| qIbHIPP7-R | CCACAACTTTGCTCGCCTTA |

| 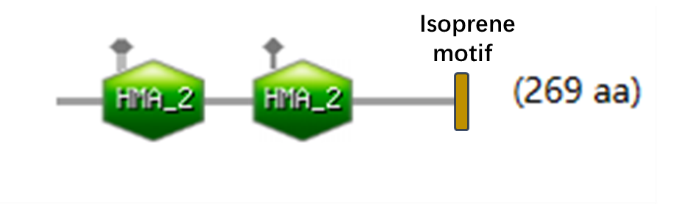  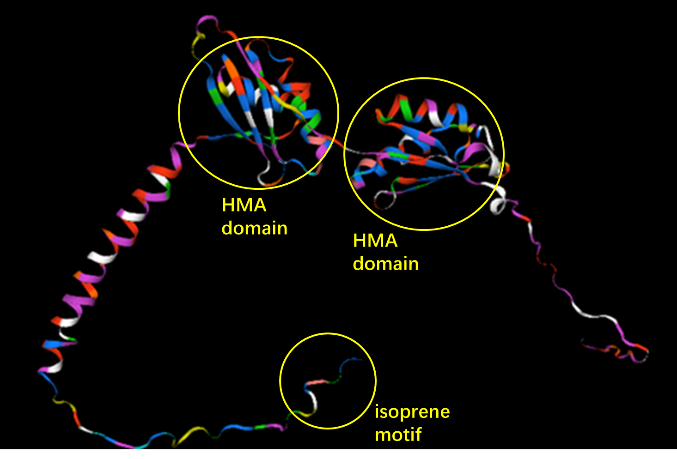 |
| --- |

**Fig. S1. Protein structure analysis of IbHIPP7.**

| **A** | **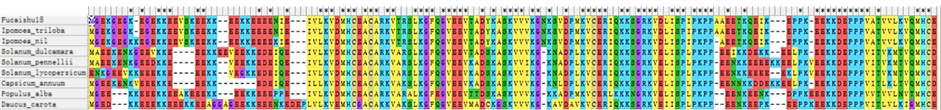** |
| --- | --- |
|  | **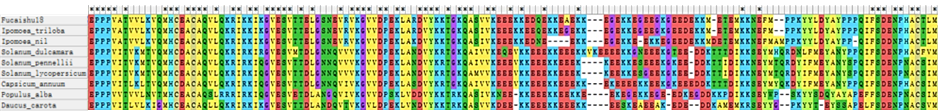** |
| **B** | **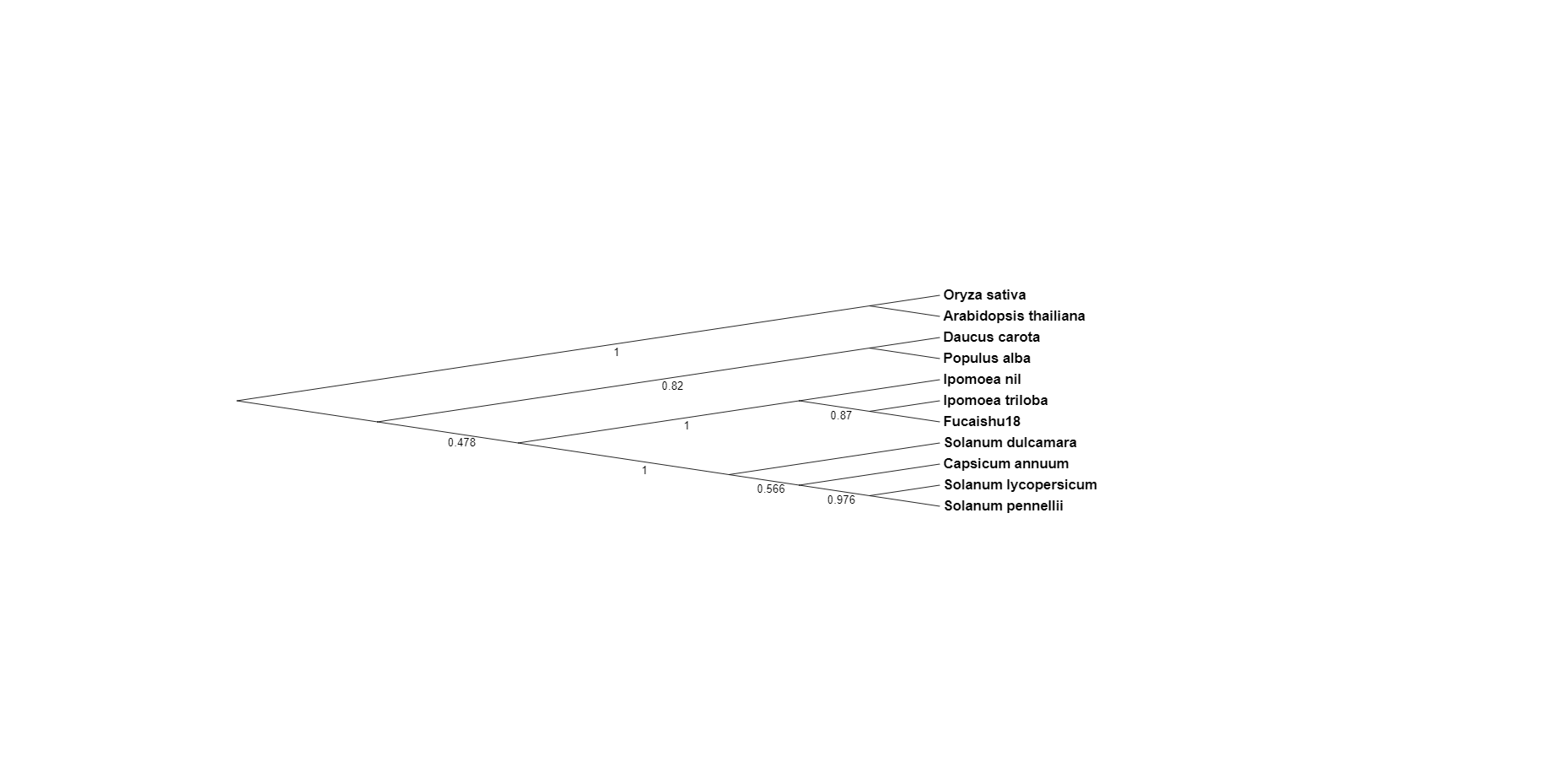** |

**Fig. S2. Phylogenetic analysis of the amino acid sequences of IbHIPP7 from Fucaishu18. (A) Sequence alignment of the amino acid sequences of HIPP7 from Fucaishu18, *Ipomoea_triloba* (ItHIPP7, XM_031241034.1), *Ipomoea_nil* (InHIPP7, XM_019323565.1), *Solanum_dulcamara* (SdHIPP7, XM_055946532.1), *Solanum_pennellii* (SpHIPP7, XM_015216304.2), *Solanum_lycopersicum* (SlHIPP7, XM_026030620.1), *Capsicum_annuum* (CaHIPP7, XM_047412400.1), *Populus_alba* (PaHIPP7, XM_035042330.1) and *Daucus_carota* (DcHIPP3, XM_017373625.1). The amino acids with the same color belong to the same class and have similar structures and functions. ‘*’ indicates that it is very conservative, and all amino acids in this position are the same. No labeling indicates that it is not very conservative, as different types of amino acids appear at this position. (B)** **The phylogenetic tree of HIPP7 in sweetpotato and other related species. The Maximum Likelihood analysis was performed using the JTT+G+F model.**

| A |  |  |  |
| --- | --- | --- | --- |
| B |  | **C** |  |

**Fig. S3. Expression patterns of *IbHIPP7*. (A) Relative expression level of *IbHIPP7* gene in roots, stems, petioles and leaves of Fucaishu18. The expression level in the roots was set to 1. (B) Expression of *IbHIPP7* in roots, stems petioles and leaves under different concentrations of CdCl_2_ treatment for 48 h. (C) Expression of *IbHIPP7* in roots, stems petioles and leaves under different concentrations of CdSO_4_ treatment for 48 h. *IbActin* was used as an internal control and gene-specific primers were used for qRT-PCR analysis.**

**
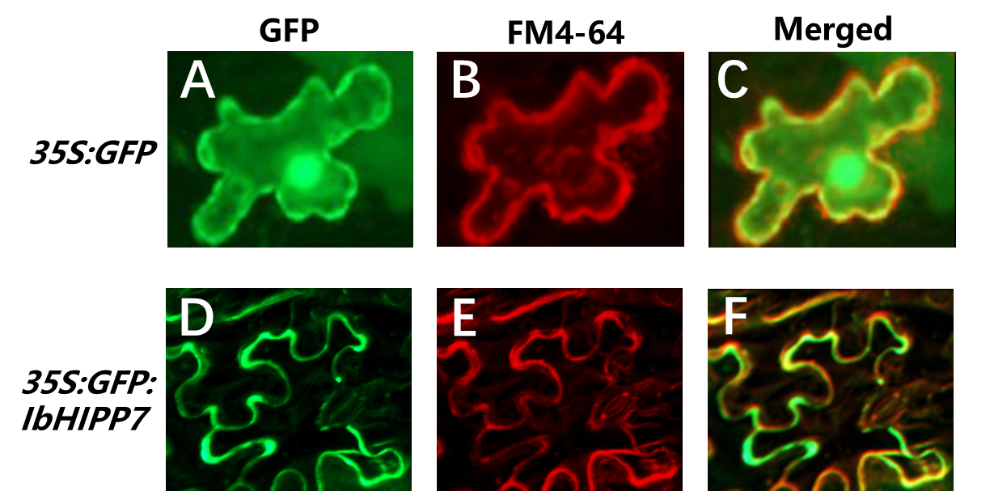
**

**Fig. S4. Subcellular localization of IbHIPP7 in tobacco leaves. (A) Green fluorescence of pCambia1305-35S: GFP empty vector. (B) Red fluorescence of FM4-64. (C) Merged fluorescence of A and B. (D) Green fluorescence of pCambia1305-35S: GFP: IbHIPP7 fusion. (E) Red fluorescence of FM4-64. (F) Merged fluorescence of D and E.**

**A**


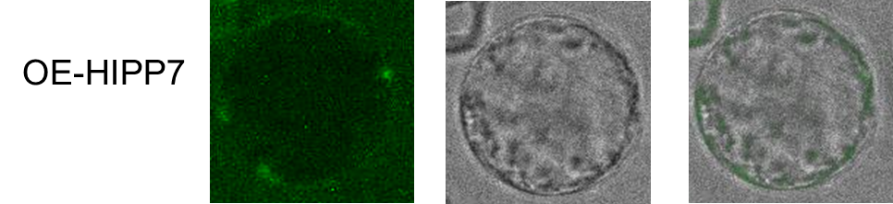


**B**

**
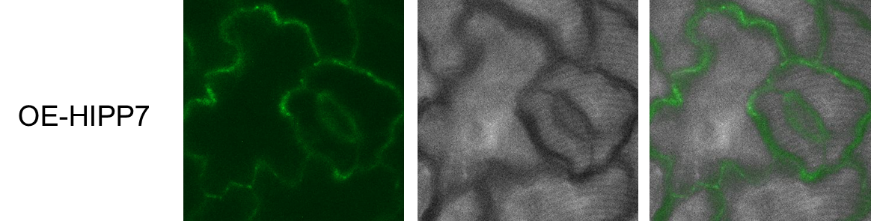
**

**Fig. S5. Subcellular Localization of IbHIPP7 in the stable transformation system. (A)** **Subcellular localization of IbHIPP7 in overexpressing sweetpotato protoplasts. (B) Subcellular localization of IbHIPP7 in overexpressing sweetpotato leaf epidermal cells.**


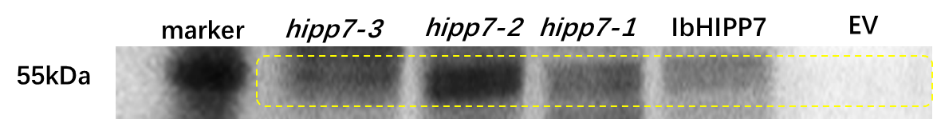


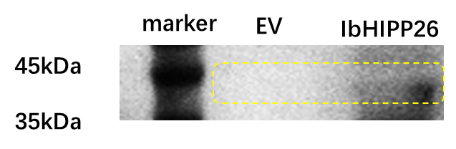


**Fig. S6. Western blot analysis of GFP-tagged IbHIPP proteins expressed in yeasts. Proteins were extracted from yeast cells induced with 2% galactose for 12 h, separated by SDS-PAGE, and transferred to PVDF membranes. The membrane was probed with an anti-GFP antibody. The positions of molecular weight markers (kDa) are indicated on the left. GFP: IbHIPP26 was detected at approximately 41 kDa, while GFP: IbHIPP7 and its domain-deletion derivative were detected between 55-57 kDa, consistent with their predicted sizes.**


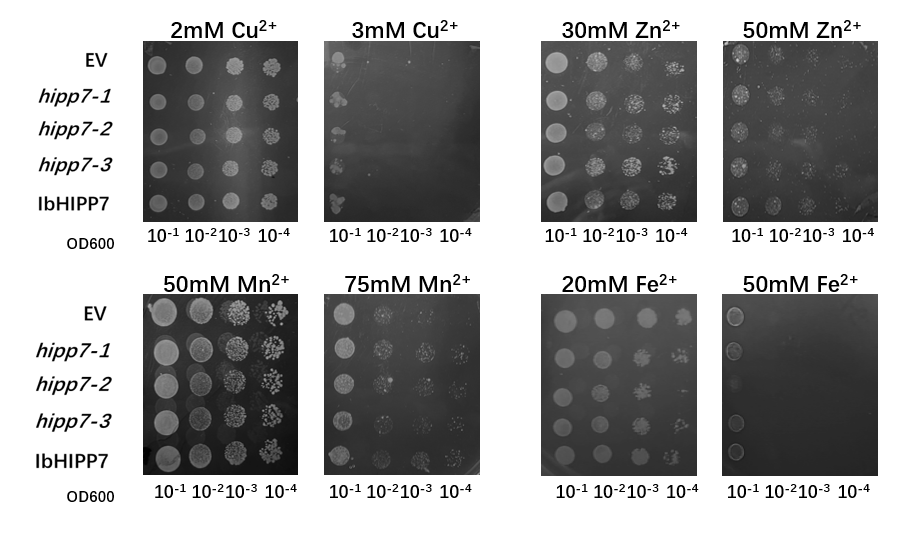


**Fig. S7. Phenotypic analysis of yeast tolerance to Cu^2+^, Mn^2+^, Zn^2+^, and Fe^2+^ following deletion of different functional domains of IbHIPP7.**

**A B**


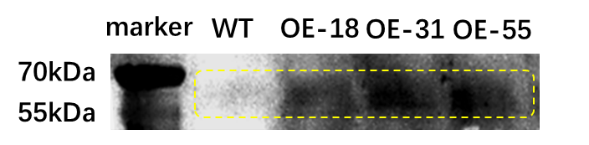


**Fig. S8. Identification of positive transgenic arabidopsis. (A) qPCR identification. The mRNA expression level of *IbHIPP7* in the WT was set to 1. (B) Western blot analysis. Proteins were extracted from leaves of arabidopsis.** **A GFP antibody specific to the fused tag was used to confirm protein expression. The positions of molecular weight markers are indicated on the left. GFP: IbHIPP7 were detected between 55–57 kDa, consistent with their predicted sizes.**


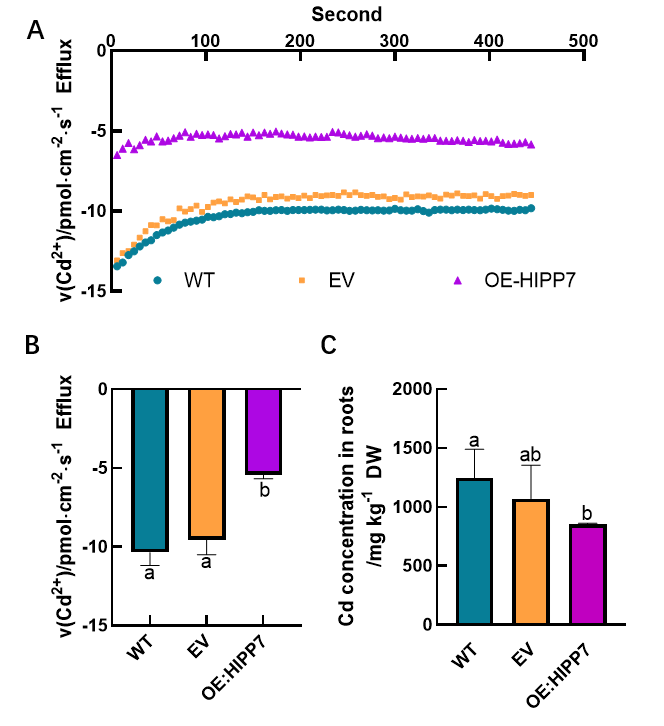


**Fig. S9. Responses of transgenic Fucaishu18 hairy roots to Cd treatment. (A) and (B) Net Cd^2+^ flux measurements in 5-mm-long root segments of Fucaishu18 hairy roots. Fluxes were detected at a position 300 μm from the root tip in three groups: WT (teal dots), *IbHIPP7*-overexpression lines (purple dots), and EV-transformed lines (orange dots). All samples were incubated in a measuring solution containing 0.1 mM KCl, 0.01 mM CdCl_2_, and 0.3 mM 2- (N-morpholino) ethanesulfonic acid (MES) (pH 5.6) during the assay. (C) Cd concentration in transgenic Fucaishu18 hairy roots. Hairy roots (WT, *IbHIPP7*-overexpression, and EV lines) were generated via *Agrobacterium rhizogenes*-mediated genetic transformation. Seedlings were first acclimated in 1/4-strength Hoagland solution for 2 weeks, then treated with 10 μM CdCl_2_ for 7 days. Roots were harvested, and Cd content was determined by ICP-MS. Each biological replicate included three seedlings.**

**A B**


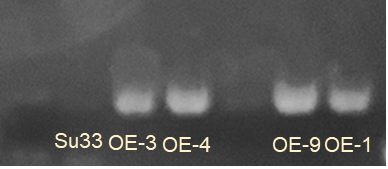

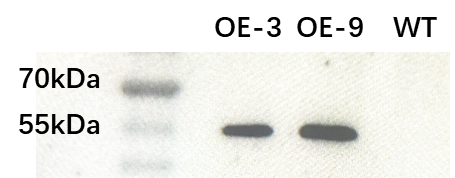


**Fig. S10. Confirmation of positive transgenic sweetpotatoes. (A). PCR identification of positive transgenic sweetpotatoes. (B). Western blot for detection of GFP-HIPP7 fusion protein in transgenic sweetpotatoes. A GFP antibody specific to the fused tag was used to confirm protein expression.**

**
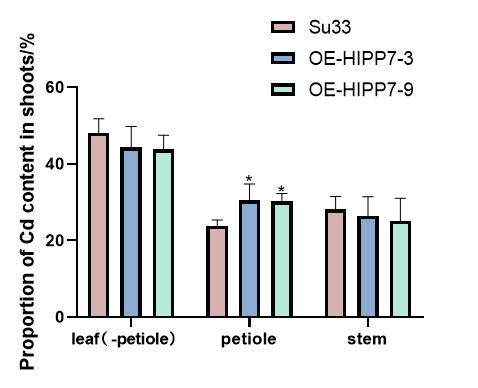
**

**Fig. S11. Proportion of Cd content in different above-ground organs relative to total Cd content in shoots.**
